# Synthesis and characterization of NiCr_2_O_3_-CeO_2NRs_ anode electrocatalyst for ethanol electrooxidation reaction in alkaline media

**DOI:** 10.55730/1300-0527.3529

**Published:** 2022-11-29

**Authors:** Emine Sena KAZAN-KAYA, Mahmut BAYRAMOĞLU

**Affiliations:** Department of Chemical Engineering, Faculty of Engineering, Gebze Technical University, Kocaeli, Turkey

**Keywords:** EOR, nonnoble, anode catalyst, alkaline media, electrocatalysts

## Abstract

In this paper, ceria (CeO_2_) nanorod (NR) supported Ni-Cr_2_O_3_ anode electrocatalysts were synthesized as nonnoble metal-based anode electrocatalysts for ethanol electrooxidation reaction (EOR) in alkaline media. Physicochemical characterization of the electrocatalysts was investigated by XRD, SEM, and TEM techniques. Electrochemical performances of the catalysts were investigated via cyclic voltammetry (CV), linear sweep voltammetry (LSV), and chronoamperometry (CA) techniques. The data from linear LSV were used for the diffusion coefficient of the electrocatalysts. The CA experiments’ results showed the tolerance for catalytic poisoning and durability of the synthesized electrocatalysts.

## 1. Introduction

The ever-increasing need for energy and depleting fossil fuels has been a motivation for researchers to search for new energy generation systems. In this context, alcohol electrooxidation reactions get attention [1–5]. Among the alcohols, ethanol which is an economical, nontoxic fuel is considered as a promising renewable alternative to fossil fuels since it has a high theoretical energy density. Direct Ethanol Fuel Cells (DEFCs) are used to produce energy from ethanol [6]. This eco-friendly system has a high energy conversion efficiency and low operating temperature; however, it is restricted for commercial applications since it generally uses noble metal-based anode electrocatalysts for ethanol electrooxidation reaction (EOR). Noble metal-based anode electrocatalysts are very expensive and have low stability. Therefore, it is significant to synthesize a nonnoble metal-based anode electrocatalyst for the EOR reaction.

The EOR is faster in alkaline media besides, transition metal-based anode electrocatalysts can be used in this system. Among the transition metals, nickel is considered as an active and stable anode catalyst compared to Pd, Pt. Moreover, its low cost and excellent ethanol oxidation performance led researchers to study nickel-containing anode catalysts. There are various studies conducted with Ni-based anode electrocatalysts for electrooxidation of ethanol in alkaline media [7]. As known, the electrocatalytic activity of nickel can be enhanced with support materials and/or cocatalysts. Chromium and chromium oxides are considered as significant cocatalysts and/or support materials. In this context, Cr_2_O_3_ is considered as an important cocatalyst material for fuel cell applications [8–11]. It is used as a promising cocatalyst for alcohol electrooxidation reactions.

For instance, Ullah et al. prepared Ni-Pd nanoparticles and used Cr_2_O_3_ as a support material for methanol electrooxidation reaction (MOR) in alkaline media. Accordingly, it was found that compared to nanoparticles supported on activated carbon and glassy carbon electrodes, Cr_2_O_3_ addition to Ni-Pd resulted in a considerable improvement in catalytic activity and tolerance for methanol oxidation [12]. Hassan and Hamid synthesized Ni-Cr_2_O_3_/C anode electrocatalyst for EOR in alkaline media. They used Cr_2_O_3_ to promote the EOR by increasing the charge transfer process in the Ni^3+^/Ni^2+^ couple transformation. They pointed out that, Cr_2_O_3_ has similar catalytic activity in alkaline solutions as Co_3_O_4_ or CuO, and the presence of Cr_2_O_3_ boosts the charge transfer process. Additionally, they reported that the addition of Cr_2_O_3_ reduces the particle size of Ni and enhances the catalytic activity towards EOR by increasing the active sites in the catalyst [13]. In an alkaline solution, the Ni^3+^ ion is thought to be the most important species for alcohol oxidation on Ni or Ni-based electrodes. Furthermore, the presence of mixed oxides may act as an electron transfer mediator for EOR. Apparently, Cr_2_O_3_ is an important cocatalyst material for fuel cell applications among transition metal compounds [14–18]. It has the potential as a cocatalyst for alcohol electrooxidation processes [19, 20]. Unfortunately, the lack of studies with Cr_2_O_3_ is a gap in the literature therefore, this gap must be filled with valuable studies.

CeO_2_ is amongst the most significant cocatalysts. Because of its good oxyphilic properties, CeO_2_ is known to aid electron transport during electrochemical processes. It has been documented in the literature that anode catalysts including CeO_2_ produce a high current density during the electrooxidation of alcohols and promote a negative onset potential shift. As known, the shape and size of nanoparticles highly affect the activity of the catalysts they form. Thus, the morphology of CeO_2_ has been proven in the literature to affect catalyst interactions. CeO_2_ nanorods are high aspect ratio nanomaterials and these nanorods have been shown to have greater oxygen vacancy density and catalytic activity for processes such as EOR [21–25].

According to the literature research, it is known that transition metal oxides will increase the electrocatalytic activity of Ni thus, this issue should be emphasized more. To our knowledge, there is no study in the literature that uses NiCr_2_O_3_-CeO_2NRs_ electrocatalysts for EOR in alkaline media. Accordingly, the aim of this study is to find the most suitable weight ratio for ethanol electrooxidation performance by synthesizing NiCr_2_O_3_-CeO_2NRs_ electrocatalysts in different weight ratios using Cr_2_O_3_ and CeO_2_ nanorods as cocatalyst and support materials. Nickel-containing catalysts were synthesized by the modified polyol method and their physicochemical characterizations were investigated by XRD, SEM-EDS, and TEM techniques. The electrochemical performances of the catalysts were observed by CV, LSV, and CA methods.

## 2. Materials and methods

### 2.1. Materials

Ethanol (Merck), NiCl_2_.6H_2_O (Sigma Aldrich), NaOH (Sigma Aldrich), Hydrazine Hydrate (64%–65%, Sigma Aldrich), Ethylene glycol (Merck), KOH (Sigma Aldrich), Cr(NO_3_)_3_.9H_2_O (Merck), Ce(NO_3_)_3_.6H_2_O (Merck). All the chemicals were used as received without further purifications.

### 2.2. Preparation of the catalysts

Cr_2_O_3_ NPs were synthesized via the thermal decomposition method [26]:

The required amount of Cr(NO_2_)_3_.9H_2_O is mixed with DI water and stirred at room temperature. The solution was dried at 110 °C for 12 h and then calcinated at 800 °C (heating rate 5 °C min^−1^) for 5h.

CeO_2_ NRs were prepared as follows [27]:

3 g of Ce(NO_3_)_3_.6H_2_O were dissolved in DI water. This solution is mixed with the required amount of 11.86 M NaOH solution (70 mL) for 30 min and then, poured into an autoclave at 100 °C for 24 h. Next, the final solution was cooled down to room temperature naturally, filtered, washed with DI water, and dried at 65 °C overnight.

Ni-Cr_2_O_3_/CeO_2NR_ electrocatalysts were synthesized via a modified polyol method [28]:

Required amounts of 0.1 M NiCl_2_.6H_2_O in ethylene glycol (EG), EG, Cr_2_O_3,_ and CeO_2_ NRs were mixed for a while then the mixture was heated to 120 °C in an oil bath. Next, a proper amount of hydrazine hydrate (HH) was added and mixed for 2 min. Lastly, 0.5 M NaOH (in EG) was added and stirred. The reaction is completed with the formation of black participate.

Preliminary experiments were carried out to determine the catalyst combinations given in Table 1. As a result of the preliminary experiments, the weight range used in the catalyst combinations was determined in Table 1. The aim here is to find the weight ratio of the catalyst with the highest EOR performance among the prepared electrocatalysts. Each catalyst was desired to contain 20% wt. CeO_2_ NR and %80 wt. NiCr_2_O_3_. In addition, it is planned that the NiCr_2_O_3_ portion of each catalyst will have different weight ratios of Ni and Cr_2_O_3_ NPs. Accordingly, NiCr_2_O_3_ - 1, NiCr_2_O_3_ -2, and NiCr_2_O_3_ -3 have 95%, 90%, and 85% Ni NPs by weight, respectively.

### 2.3. Morphological characterization

The X-ray diffraction (XRD) analyses were performed with Bruker D8 Advance device (40Kw, 40mA), with a Cu Kα radiation over a 2θ range from 2°–90° and with 3° min^−1^ scanning rate. Microstructures of samples were investigated by SEM analysis (XL-30 SFEG, Philips), TEM analysis was made with FEI TALOS F200S, XPS analysis (Phoibos 150 Specs).

### 2.4. Electrochemical characterization

The electrocatalytic activity of the NiCr_2_O_3_-CeO_2NRs_ electrocatalysts towards ethanol oxidation reaction (EOR) was performed by CHI 1100C Potentiostat using a glassy carbon electrode (GCE) with an area of 0.07cm^−2^ as working electrode, a Pt wire as the counter electrode, and lastly an Ag/AgCl electrode as a reference electrode.

Nickel-based anode electrocatalysts were activated in 0.1M KOH solution at 0.01 Vs^−1^ scan rate. CV, LSV, and CA experiments were performed in a solution containing 0.05M KOH and 0.5M CH_3_CH_2_OH.

## 3. Results and discussion

### 3.1. Morphological characterization results

**Figure 1:**
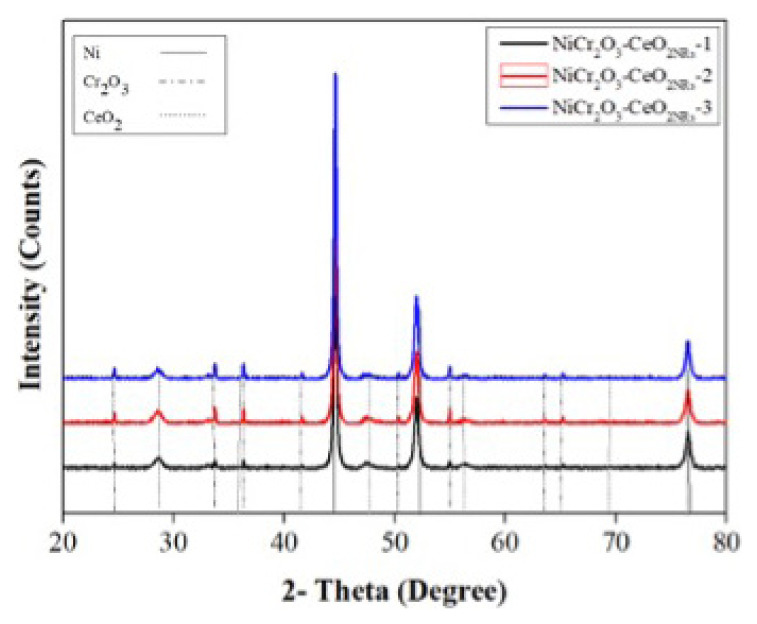
XRD results of NiCr_2_O_3_-CeO_2NRs_ electrocatalysts.

The XRD results of the synthesized catalysts are exhibited in Figure 1. Accordingly, the peaks observed at 2θ = 24.5, 33.7, 36.3, 39.8, 41.5, 44.3, 50.31, 54.9, 58.4, 63.5, 65.2, 73.0, 76.9, 79.1 in Figure 1 were the (012), (104), (110), (006), (113), (202), (024), (116), (122), (214), (300), (119), (220), (306) surfaces of Cr_2_O_3_, respectively [29]. Nickel (111), (200), and (220) surfaces were observed at 2 = 44.32, 51.74, and 76.95 [12]. The surfaces of CeO2 (111), (200), (220), (311), (222), (400), (331), and (420) are shown by the peaks at 2 = 28.60, 33.14, 47.55, 56.39, 59.16, 69.49, 76.79, 79.14, respectively [30]. Cr_2_O_3_ peaks are more prominent than CeO_2_ peaks. Figure 1 shows the XRD findings of NiCr_2_O_3_-CeO_2NRs_ electrocatalysts. All of the components’ peaks are clearly selectable, and there are no peaks visible outside of them. As a result, binary catalysts may plainly be observed to be effectively synthesized.

**Figure 2:**
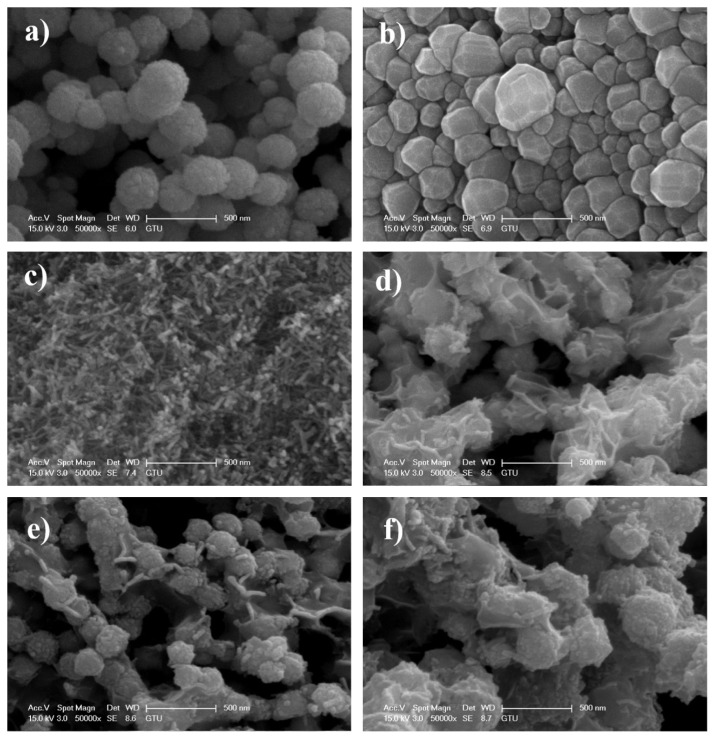
SEM results of a) Ni NPs, b) Cr_2_O_3_ NPs_,_ c) CeO_2_ NRs, d) NiCr_2_O_3_-CeO_2NRs_ −1, e) NiCr_2_O_3_-CeO_2NRs_ −2, f) NiCr_2_O_3_-CeO_2NRs_ −3.

The SEM results of the Ni NPs, Cr_2_O_3_ NPs, CeO_2_ NRs, and NiCr_2_O_3_-CeO_2NRs_ electrocatalysts are given in Figure 2. Characteristic structures of the Ni NPs, Cr_2_O_3_ NPs, CeO_2_ NRs can be selected clearly from Figure 2a–2c and therefore, it can be concluded that the Ni NPs, Cr_2_O_3_ NPs, CeO_2_ NRs are well-synthesized. Figure 2d–2f represents the SEM results of NiCr_2_O_3_-CeO_2NRs_ electrocatalysts. It is seen that the surface of the NiCr_2_O_3_ NPs was covered with CeO_2_ NRs well.

**Figure 3:**
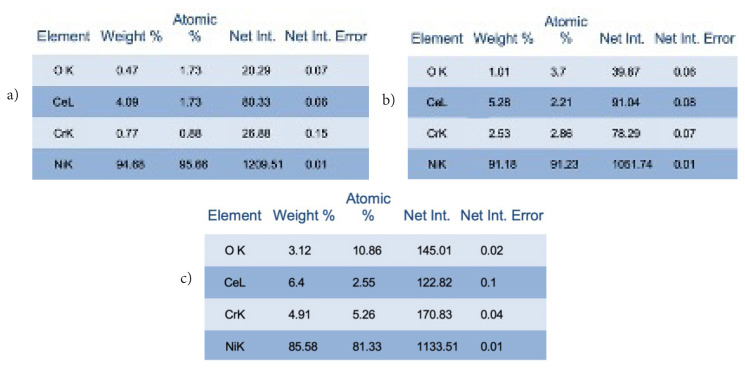
EDS analysis results of a) NiCr_2_O_3_-CeO_2-NRs_ −1, b) NiCr_2_O_3_-CeO_2-NRs_ −2, c) NiCr_2_O_3_-CeO_2-NRs_ −3.

The composition of the synthesized catalysts was investigated via EDS analysis, and the results are displayed in Figure 3. Here, it is seen the EDS results of the NiCr_2_O_3_-CeO_2-NRs_ −1, NiCr_2_O_3_-CeO_2-NRs_ −2, NiCr_2_O_3_-CeO_2-NRs_ −3. The corresponding elements namely, Ni, Cr, Ce, and O can be detected in the prepared samples. Accordingly, the metallic Nickel atomic ratio of the NiCr_2_O_3_-CeO_2-NRs_ −1, NiCr_2_O_3_-CeO_2-NRs_ −2, NiCr_2_O_3_-CeO_2-NRs_ −3 was determined as 95.66%, 91.23%, and 81.33%, respectively.

**Figure 4:**
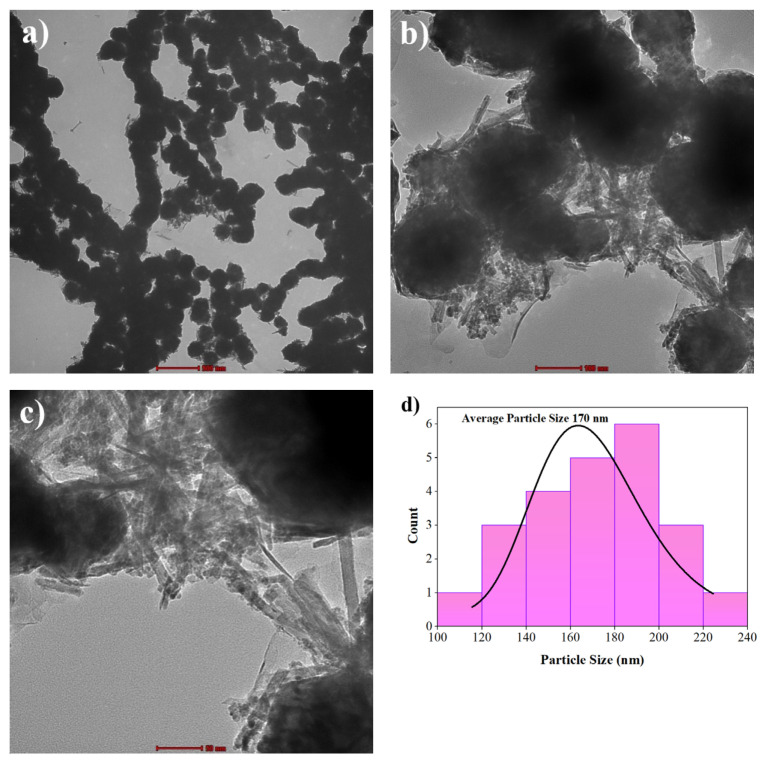
TEM results of NiCr_2_O_3_-CeO_2NRs_ −2 a) 500 nm, b) 100 nm, c) 50 nm, d) graph of the particle size distribution of NiCr_2_O_3_-CeO_2NRs_ -2.

The findings of the TEM study of the catalysts, which was carried out to gain more about the structural morphology and particle size distribution of the electrocatalysts, are displayed in Figure 4. Due to the very small size and low weight fraction, individual CeO_2_ NRs on NiCr_2_O_3_ NPs could not be distinguished in Figure 4a; exclusively CeO_2_ NRs can be seen when TEM images are magnified (see Figure 4b–4c). Figure 4d shows the average particle size distribution for NiCr_2_O_3_-CeO_2NRs_ −2.

### 3.2. Electrochemical characterization results

Catalyst ink was prepared and applied to a glassy carbon electrode (GCE) for electrochemical analysis. To prepare the ink 200 μL of isopropyl alcohol, 10 μL of Nafion solution (5 wt%), and 1 mg of catalyst were ultrasonicated for 30 min. Also, the surface area of the GCE is 0.07 cm^2^ and in order to load 1 mg cm^−^^2^ catalyst, 15 μL of the catalyst ink was attached to the GCE. The GCE’s surface was cleaned with Al_2_O_3_ paste, washed with DI water, and dried under N_2_ gas before the ink was deposited.

Then, using CHI 1100C Potentiostat, electrochemical data were measured. Before each experiment, N_2_ gas was circulated through the solutions.

**Figure 5:**
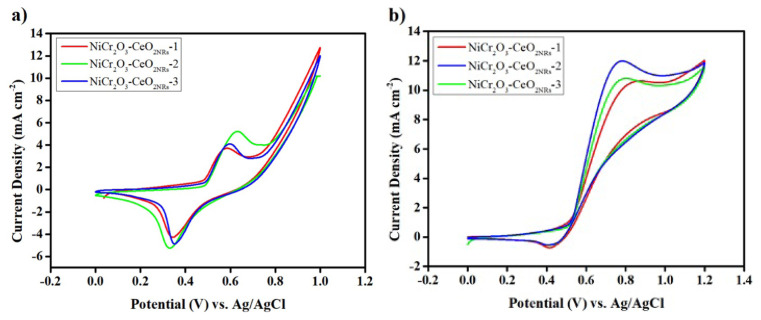
CV voltammograms of NiCr_2_O_3_-CeO_2NRs_ a) in 0.05 M KOH at 0.01 V s^−1^, b) in 0.05 M KOH + 0.5 M ethanol at 0.01 V s^−1^.

CV results are shown in Figure 5 for the synthesized electrocatalysts. CV voltammograms of the electrocatalysts were obtained at a scan rate of 0.01 V s^−1^ for electrolytes containing 0.1M KOH and 0.05M KOH+0.5M Ethanol.

The onset potential and current density are the two prominent parameters that characterize the activity of electrocatalysts. Complete ethanol oxidation to CO_2_ is a necessary condition for achieving the highest current. Figure 5a indicates that in the absence of ethanol, two redox peaks at 580 mV (anodic peak) and 450 mV (cathodic peak) were seen on the synthesized catalysts. These peaks are attributable to the nickel hydroxide (Ni(OH)_2_)/nickel oxo hydroxide (NiO(OH)) transition [31].

A rise in current density and/or the occurrence of an anodic peak indicating ethanol electrooxidation are two essential signs of the electrocatalyst’s activity towards ethanol electrooxidation. With the addition of ethanol to the blank media, the obtained current density for each of the synthesized catalysts was enhanced. Depending on the weight percentage of Cr_2_O_3_ NPs were present in the structure, the peak intensity varied. Consequently, introducing Cr_2_O_3_ NPs increased the synergistic effect and showed broad oxidation peaks.

Among the synthesized catalysts illustrated in Figure 5b, the NiCr_2_O_3_-CeO_2NRs_-2 electrocatalyst has the highest current density. Additionally, the catalysts’ onset potentials were examined. As a consequence, adding more Cr_2_O_3_ NPs until a proper amount caused the onset potentials to move negatively, indicating better catalytic activity. This increase in current density confirmed findings that the amount of Cr_2_O_3_ NPs and Ni seeks to enhance EOR. Because the amount of Cr_2_O_3_ NPs in the catalyst is crucial for maximizing the synergistic effect since too many Cr_2_O_3_ NPs may obstruct the catalyst’s active sites and reduce the synergistic effect, which will ultimately affect the electrocatalyst’s activity and durability.

In conclusion, NiCr_2_O_3_-CeO_2NRs_ electrocatalysts are beneficial at EOR, and the suitable participation of Cr_2_O_3_ has increased anodic peak current density. As can be observed from the CV tests, the electrocatalysts’ various metallic nickel weight percentages mainly generated diverse catalytic activity. It is noticeable that the catalytic activity is accelerated with a cocatalyst, even if an increase in the quantity of metallic nickel in the electrocatalyst structure does not imply an enhancement in the electrocatalytic activity. It was noticed that the amount of metallic nickel had greatly changed the catalytic activity of the synthesized catalysts and that there was a link between the maximum current density and the amount of metallic nickel in the catalyst.

The electrocatalyst’s overpotential, or the point at which the current density increases as a result of EOR, is known as the onset potential. It is crucial for an effective anode electrocatalyst to have a low onset potential and high current density since a superior electrocatalyst may be detected with lower overpotential. The overpotential is reduced when the cocatalyst is added to the electrocatalyst structure. Accordingly, the onset potentials and maximum current densities of the NiCr_2_O_3_-CeO_2NRs_-1, NiCr_2_O_3_-CeO_2NRs_ −2, and NiCr_2_O_3_-CeO_2NRs_ −3 are given in Table 2.

**Figure 6:**
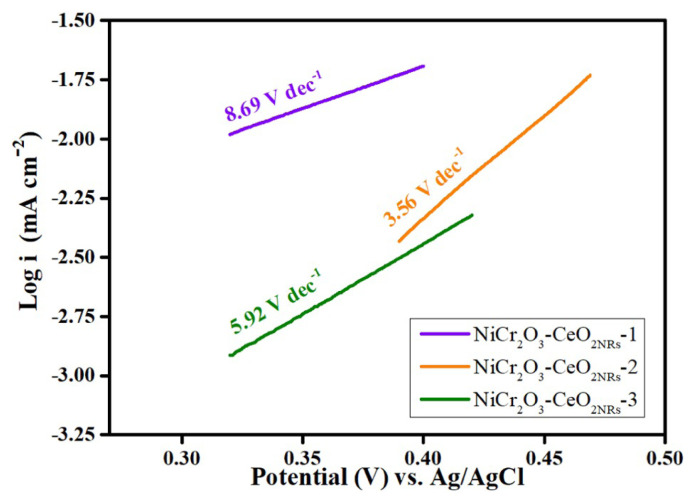
Tafel plots of NiCr_2_O_3_-CeO_2NRs_ −1, NiCr_2_O_3_-CeO_2NRs_ −2, NiCr_2_O_3_-CeO_2NRs_ - 3, respectively.

Anodic Tafel plots of the synthesized catalysts are given in Figure 6. To derive the Tafel plots, the LSV technique was used, and the curves were recorded at a sweep rate of 1 mV s^−^^1^ in 0.05 M KOH + 0.5 M ethanol solution. The Tafel curves presented in Figure 6 showed that NiCr_2_O_3_-CeO_2NRs_-2 had the lowest slope values (3.56 V dec^−^^1^), implying a lower overpotential and better reaction kinetics for oxidizing ethanol among the synthesized catalysts. The NiCr_2_O_3_-CeO_2NRs_-2 surface’s electron-transfer kinetics for EOR are better compared to other electrocatalysts according to the lower Tafel slope values. The Tafel slope’s decrease suggests an improvement in the EOR’s kinetics and could possibly predict an earlier C-C bond cleavage. The transfer coefficient (a) of NiCr_2_O_3_-CeO_2NRs_-1, NiCr_2_O_3_-CeO_2NRs_-2, and NiCr_2_O_3_-CeO_2NRs_-3 for electrooxidation of ethanol was determined to be 0.816, 0.925, and 0.875, respectively where the Tafel slope being equal to n(1-a)F/2.303 RT [32].

**Figure 7:**
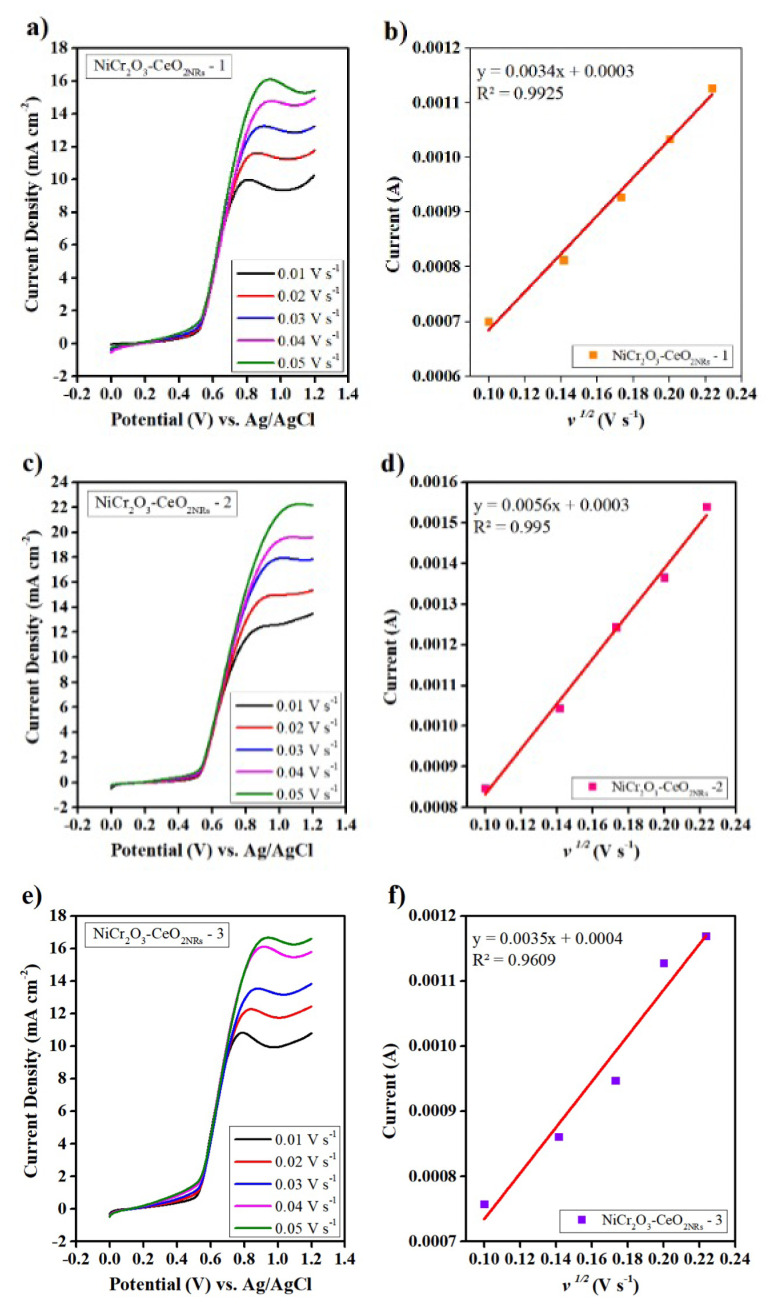
(a–b) LSV results and i_p_ vs. v^1/2^ graphs of NiCr_2_O_3_-CeO_2NRs_ −1, (c–d) LSV results and i_p_ vs. v^1/2^ graphs of NiCr_2_O_3_-CeO_2NRs_ - 2, (e–f) LSV results and i_p_ vs. v^1/2^ graphs of NiCr_2_O_3_-CeO_2NRs_ - 3, respectively.

Figure 7 displays the catalysts’ LSV experiment results. Greater current density resulted from a higher scan rate, and this correlation enables us to determine the catalysts’ diffusion coefficients. The diffusion coefficients are determined using the Randles-Sevcik formula shown below. A graph of i_p_ vs. v^1/2^ is created in order to interpret this equation.


(1) $${\text{i}}_{\text{p}}=0.4463\text{nFAC}{\left(\frac{\text{nF}v\text{D}}{\text{RT}}\right)}^{1/2}$$

Here, the i_p_ is peak current density, n is the number of electrons transferred (2.8), F is Faraday constant, A is electrodes geometric surface area, C is concentration, v is scan rate, D is diffusion coefficient, R is universal gas constant and T is temperature [33].

The role of Cr_2_O_3_ NPs in advancing the Ni catalyst toward the electrooxidation process is supposed to be well addressed by the calculation of several kinetic parameters. Accordingly, the diffusion coefficient is one of the most important parameters. NiCr_2_O_3_-CeO_2NRs_ -1, NiCr_2_O_3_-CeO_2NRs_ -2, and NiCr_2_O_3_-CeO_2NRs_ -3 electrocatalysts have diffusion coefficients of 6.31 × 10^−9^ cm^2^ s^−1^, 1.62 × 10^−8^ cm^2^ s^−1^, and 6.31 × 10^−9^ cm^2^ s^−1^, respectively. For EOR in alkaline media, Hassan and Abdel-Hamid determined the diffusion coefficient for Ni-Cr_2_O_3_/C as 6.2 × 10^−13^ cm^2^s^−1^. [14] Barakat et al. found diffusion coefficients for Ni-CNFs as 3.05 × 10^−10^ cm^2^ s^−1^.[34] Wang et al., reported that the diffusion coefficient of spherical Ni(OH)_2_ microencapsulated by cobalt NPs is 1.2 × 10^−^^9^ cm^2^ s^−^^1^. [35] Our findings outperform those found in the literature, and the NiCr_2_O_3_-CeO_2NRs_-2 electrocatalyst has the greatest diffusion coefficient among all those tested. Increased reaction kinetics result from an increase in the diffusion coefficient, which simplifies diffusion control.

**Figure 8:**
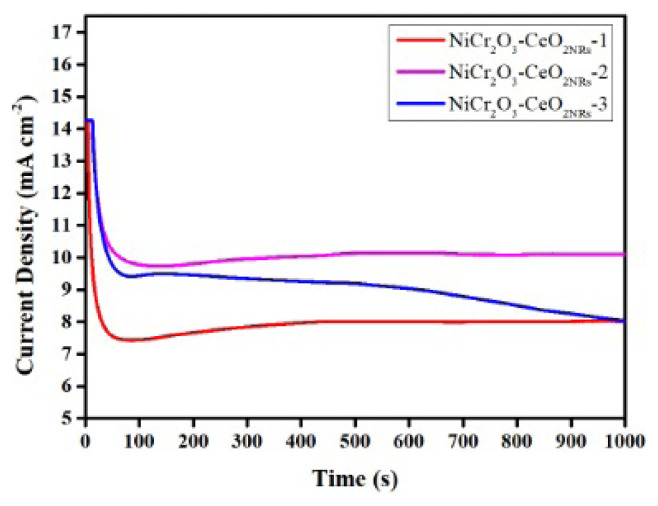
CA test results for NiCr_2_O_3_-CeO_2NRs_-1, NiCr_2_O_3_-CeO_2NRs_–2, and NiCr_2_O_3_-CeO_2NRs_-3.

The data of CA testing to examine the catalysts’ endurance are shown in Figure 8. Since the CHI 1100C potentiostat only permits 1000s for CA testing, the time frame was 1000 s. Chronoamperometry tests for the synthesized catalysts were conducted and the results are given in Figure 8. It is clear from the CV tests that the ErCi (Reversible Electron Transfer Followed by an Irreversible Homogeneous Chemical Reaction) mechanism may be involved in the EOR process [36]. As a result, under that scenario, undesirable intermediates may occur; these intermediates may even be toxic. Since it is common knowledge that poisoning intermediates can reduce catalytic activity when they are adsorbed. Therefore, the antipoisoning capability of a high-performance catalyst is crucial. Different metals and oxides may be used in catalysts to avoid catalyst poisoning. For instance, Wang et al., synthesized Pt-MO_x_/C (M= Ce, Zr) electrocatalysts and conducted a CA experiment to investigate the stability of the synthesized electrocatalysts for 1000 s in 0.1M H_2_SO_4_ + 1.0M C_2_H_5_OH solution. Accordingly, they reported that the stability of Pt-CeO_2_-ZrO_2_ (Ce:Zr = 2:1) is the best among the synthesized catalysts [37]. Another study was made by Chai et al., and they prepared Ir_3_Sn–CeO_2_/C electrocatalyst for EOR. Their CA results indicated that the stability of Ir_3_Sn–CeO_2_/C is better than Ir_3_Sn/C, Ir/C, and Pt/C [38]. Accordingly, the stability of NiCr_2_O_3_-CeO_2NRs_ -2 is better than the NiCr_2_O_3_-CeO_2NRs_ −1 and NiCr_2_O_3_-CeO_2NRs_ −3. These results are coherent with the CV and LSV tests. Therefore, it can be concluded that the resistance to catalytical poisoning of NiCr_2_O_3_-CeO_2NRs_ −2 is better among the synthesized catalysts. On the other hand, it was seen that the stability of NiCr_2_O_3_-CeO_2NRs_ −3 is very poor. It can be a result of covered active sites of the catalysts or agglomerated particles. Accordingly, the importance of the catalyst composition is proven.

## 4. Conclusion

NiCr_2_O_3_-CeO_2NRs_ anode catalysts were prepared successfully, physicochemical, and electrochemical characterizations of the catalysts were investigated. The effects of Cr_2_O_3_ NPs added to the structure of the catalyst on the current density were observed. The intensity of the peaks observed during the electrochemical characterizations changed depending on the weight percent of the Cr_2_O_3_ NPs in the structure and the NiCr_2_O_3_-CeO_2NRs_ −2 electrocatalyst had the highest current density. When the onset potentials of the synthesized catalysts were examined, it was reported that adding an appropriate amount of Cr2O3 NP showed better catalytic activity by causing the onset potentials to shift in the negative direction. However, the increase in the amount of Cr_2_O_3_ NPs in the synthesized electrocatalysts can clog the active sites of the Ni catalyst after a certain point, thus reducing the activity and durability of the electrocatalyst. This situation is most clearly seen in CA experiments. In conclusion, NiCr_2_O_3_-CeO_2NRs_ electrocatalysts are useful in EOR and appropriate incorporation of Cr_2_O_3_ increased the anodic peak current density.

## Figures and Tables

**Table 1:** Weight ratios of the components in catalyst combinations.

	NiCr_2_O_3_-CeO_2NRs_-1	NiCr_2_O_3_-CeO_2NRs_-2	NiCr_2_O_3_-CeO_2NRs_-3
Ni (wt%)	76	72	68
Cr_2_O_3_(wt%)	4	8	12
CeO_2_ NR (wt%)	20	20	20

**Table 2:** The onset potentials and maximum current densities of the NiCr_2_O_3_-CeO_2NRs_-1, NiCr_2_O_3_-CeO_2NRs_ -2, and NiCr_2_O_3_-CeO_2NRs_ -3.

	Onset potential (mV)	Maximum current density (mA cm^−2^)
NiCr_2_O_3_-CeO_2NRs_-1	519	10.6
NiCr_2_O_3_-CeO_2NRs_-2	508	12
NiCr_2_O_3_-CeO_2NRs_-3	515	10.8
